# The Cost of Provisions in Institutions.—XIII

**Published:** 1904-07-23

**Authors:** 


### HOSPITAL ADMINISTRATION.

### CONSTRUCTION AND ECONOMICS.

### THE COST OF PROVISIONS IN INSTITUTIONS.—XIII.

By the Author of "Hospital Expenditure—The Commissariat."

(*Continued from page* 284.)

SPECIMEN FORMS.

The art of keeping a separate account of the expenditure on each section of the hospital household depends on a proper use of the material in the shape of estimates and accounts common to all well-managed households.

The procedure is somewhat as follows:—

1. A small diet-sheet is filled up daily by the Sister of each ward, on which the diets and extras ordered for each patient are entered.

2. These diet-sheets are combined by the housekeeper and entered on a larger diet-sheet, which, when it is filled in, shows the day's diet for all the patients in the hospital.

3. By the help of form No. 2 the housekeeper can now compile her daily requisition form, which will show in separate columns the amount required under various items for patients, medical officers, nurses, and house. This requisition-form, daily filled up by the housekeeper after inspection of what remains over from the day before, is the hinge upon which the whole system turns, and when the housekeeper has been trained to prepare it with rigid accuracy half the battle of economy is won.

Too much stress cannot be laid upon the importance of this form. The items enumerated will vary naturally according to the requirements of the institution, and may very probably run into a good many more headings than those enumerated. The column relating to patients will be filled in mechanically from the diet-sheet already before the housekeeper. But the three other columns will require some consideration and planning out. After a few weeks of experience, however, even a weak housekeeper will get a firmer grip on the reins, through this daily dissection of household requirements. We may suggest, for the convenience of such housekeepers as desire to transfer the remains of one table to another, that a note of such transactions should appear at the bottom of this sheet, as, for instance, under "household," might be noted: cold joints from officers' table, 14 lbs." In the succeeding step in the accounts, which we have now to detail, this note would find its due place, and the balance of expenditure be properly adjusted.

We append a specimen form which will be found suitable for this purpose.

Form I.—*Housekeeper's Daily Requisition Sheet.*

| — | | Patients | Medical Officers | Nurses | House- |
|---|---|---|---|---|---|
| Meat.. .. | 1. Beef .. .. | | | | |
| | 2. Mutton .. .. | | | | |
| | 3. Shin for Beef-tea, etc. .. .. | | | | |
| Fish .. .. | 1. Ordinary Supplies | | | | |
| | 2. Soles .. .. | | | | |
| | 3. Oysters .. .. | | | | |
| Poultry .. | 1. Fowls .. .. | | | | |
| | 2. Rabbits, etc. .. | | | | |
| Bread, Flour, etc... .. | 1. Bread .. .. | | | | |
| | 2. Flour .. .. | | | | |
| | 3. Oatmeal .. .. | | | | |
| | 4. Cake .. ... | | | | |
| Vegetables and Fruit.. | 1. Potatoes .. .. | | | | |
| | 2. Other Vegetables.. | | | | |
| | 3. Fruit .. .. | | | | |
| Milk and Cream .. | 1. Milk .. .. | | | | |
| | 2. Cream .. .. | | | | |
| Cheesemongery | 1. Butter .. .. | | | | |
| | 2. Cheese .. .. | | | | |
| | 3. Ham and Bacon .. | | | | |
| Eggs .. .. | 1. Cooking .. .. | | | | |
| | 2. New-laid .. .. | | | | |
| Grocery .. | 1. Tea... .. .. | | | | |
| | 2. Coffee .. .. | | | | |
| | 3. Cocoa .. .. | | | | |
| | 4. Jams, etc... .. | | | | |
| Aerated Waters .. | Aerated Waters | | | | |
| Alcohol .. | Various Headings .. | | | | |

4. From the housekeeper's daily requisitions can now be compiled the weekly summary of diets for the whole hospital. This may be prepared by the steward, secretary, matron or housekeeper, according to the size of the institution. It gives the amounts consumed under each heading by each section of the household. By means of this form there should be no difficulty in reaching the fifth

Form II.—*Week ending* .................., 190.... *Average No. of Patients* .......... *No. of Nurses* ..........

| Name | Amount | Goods Supplied | New Supplies | Brought Forward | Total | Patients | Nurses | Medical Officers | House | Total | Carried Forward |
|---|---|---|---|---|---|---|---|---|---|---|---|
| | | Meat, lb .. (Here follows list of items.) | | | | | | | | | |
| Total ..£ | | | | | | | | | | | |

Examined by ............. Alcohol for Patients ...... per week.
" " Nurses ...... "
" " House ...... "
.................. Steward.

Average cost of each Patient .......... per week.
" " " Nurse .......... "
" " " R.M.O. .......... "

step, which is to reveal the weekly cost of boarding every member of the household.

5. There are more ways than one of making out the weekly *résumé* of the cost and quantity of provisions bought and consumed. We append two forms, both of which are excellent. The first is in use in the Royal Free Hospital, and we understand from Mr. Thies that the preparation of this weekly statement to lay before the Board is about a day's work for one clerk.

The next form is adapted from one in use at the Great Northern Central Hospital. The subdivision of the inmates is somewhat different, but the principle is the same.

The excellent system in use at St. Mary's Hospital combines the daily details of consumption and cost with the weekly summary, and is too large for reproduction here. We have chosen the forms in use at institutions of moderate size, believing that they are more likely to be of service.

Form III.—*Account of Provisions Purchased and Consumed for Week ending*..........................190...

| — | Stock Consumed | | | | | | | | | Stock Purchased | | | Brought forward | Total Stock | Remaining in Stock |
|---|---|---|---|---|---|---|---|---|---|---|---|---|---|---|---|
| | Patients | | Medical Officers | | Nurses | | Household | | Total | | | | | | |
| | Quantity | Value | Quantity | Value | Quantity | Value | Quantity | Value | Quantity | Quantity | Rate | Value | Quantity | Quantity | Quantity |
| MEAT<br>Beef, etc. lb.<br>(Here follows list of items) | | | | | | | | | | | | | | | |
| Total cost for week | | | | | | | | | | | | | | | |

Diets (No. of Patients).

| — | S. | M. | T. | W. | T. | F. | S. | Total |
|---|---|---|---|---|---|---|---|---|
| Admission.. | | | | | | | | |
| Ordinary .. | | | | | | | | |
| Children .. | | | | | | | | |
| Total .. | | | | | | | | |
| Pay patients | | | | | | | | |

| — | S. | M. | T. | W. | T. | F. | S. | Total |
|---|---|---|---|---|---|---|---|---|
| No. R.M.O.'s .. | | | | | | | | |
| „ Other Officers | | | | | | | | |
| „ Nurses .. | | | | | | | | |
| „ Servants (male) | | | | | | | | |
| „ Servants (female) | | | | | | | | |
| Total .. .. | | | | | | | | |

Examined

............................................

Secretary.

............................................

Clerk.

